# Endothelial dysfunction in patients with type 2 diabetes: the truth is in the blood

**DOI:** 10.1172/JCI193128

**Published:** 2025-05-15

**Authors:** Sarah Costantino, Shafeeq A. Mohammed, Francesco Paneni

**Affiliations:** 1 Center for Translational and Experimental Cardiology (CTEC), Department of Cardiology, University Hospital Zurich and University of Zurich, Zurich, Switzerland.; 2 University Heart Center, Cardiology, University Hospital Zurich, Zurich, Switzerland.

## Abstract

Endothelial dysfunction remains a cornerstone of diabetic vascular complications. RBCs emerge as pivotal players in endothelial dysfunction, yet the underlying mechanisms remain elusive. In this issue of the *JCI*, Collado et al. show that the detrimental action of RBCs on the endothelium is mediated by extracellular vesicles (EVs). EVs derived from RBCs (RBC-EVs) of patients with diabetes were taken up by the endothelium and were able to impair endothelium-dependent relaxation via an EV-mediated transfer of the prooxidant enzyme arginase-1 (Arg1) from RBCs to endothelial cells. These findings reveal events implicated in vascular oxidative stress and set the stage for personalized approaches preventing RBC-EVs’ uptake by the endothelium.

## Finding the culprit in diabetic endothelial dysfunction

Endothelial dysfunction is a well-known precursor of atherosclerotic vascular disease and an independent predictor of morbidity in patients with diabetes (1). Experimental work conducted over the last decades has identified several mechanisms linking high blood glucose levels with the activation of signaling pathways of endothelial damage including polyol and hexosamine flux, advanced glycation end products (AGEs), protein kinase C (PKC) activation, and NF-κB–mediated vascular inflammation (1, 2). These pathways have been shown to converge on ROS generation in the vascular endothelium, thus making oxidative stress an attractive therapeutic target to tackle diabetic vascular disease (3). However, antioxidant therapies targeting both cytosolic and mitochondrial ROS have failed to improve outcomes in patients with cardiovascular disease (4). Hence, our comprehension of the mechanisms underpinning ROS generation and the specific sites where ROS exert their detrimental action as well as the dynamics involved in oxidative stress accumulation remain scarce. Unveiling the chain of events implicated in vascular oxidative stress may set the stage for mechanism-based therapeutic strategies in patients with diabetes.

## RBCs on stage

**Figure 1:**
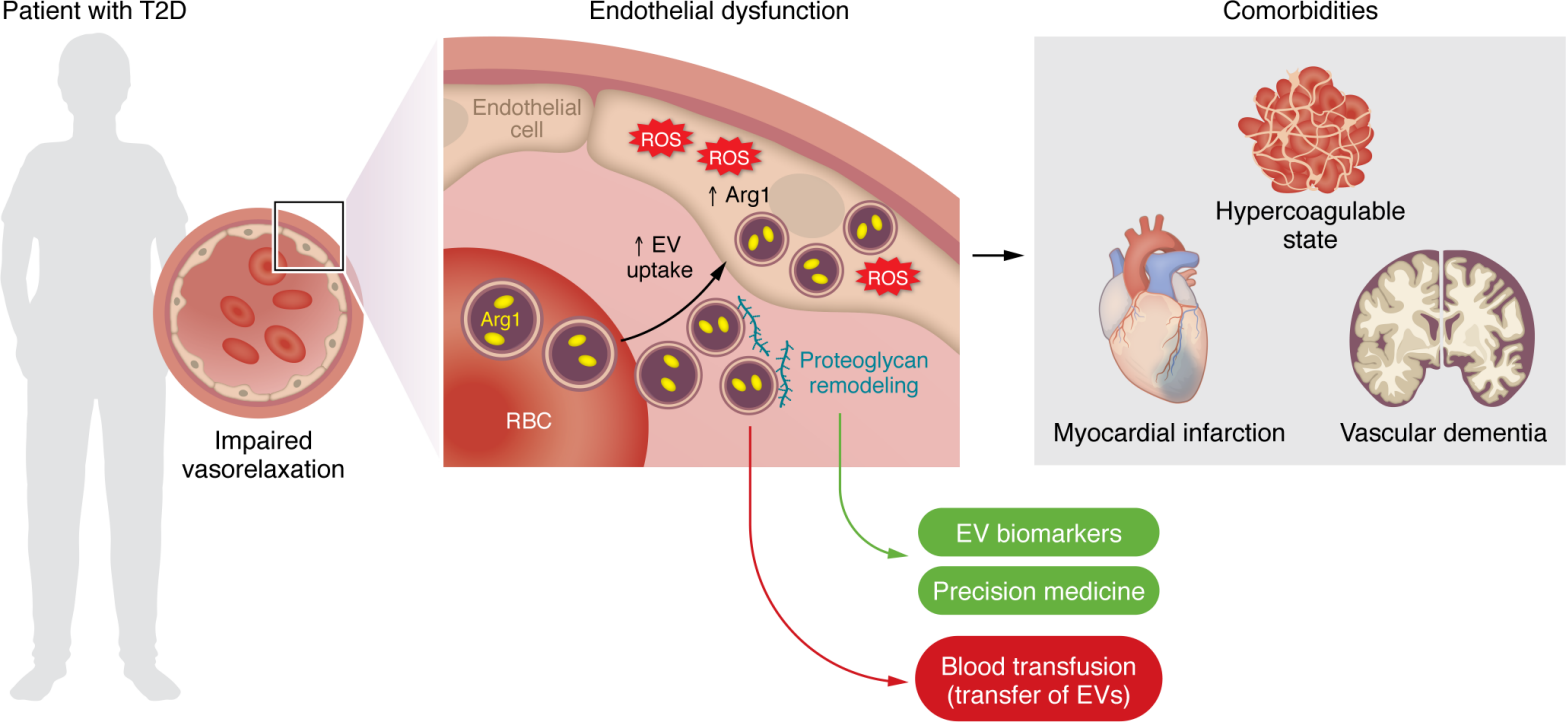
RBC-derived EVs cause endothelial dysfunction in diabetes. EVs derived from RBCs of patients with T2D are taken up by the endothelium and impair endothelium-dependent relaxation via an EV-mediated transfer of the prooxidant enzyme Arg1. Proteoglycan remodeling is a main event fostering EV uptake and may provide targets that could serve as disease biomarkers and/or personalized therapies for blocking EV uptake. RBC-EVs are also implicated in diabetes-related comorbidities, namely hypercoagulability, myocardial infarction, and vascular dementia. Blood transfusion from patients with cardiovascular risk factors represents a potential issue given the transfer of RBC-EVs and subsequent endothelial dysfunction in the recipient patient.

Recent work has revealed an unprecedented role of RBCs in diabetic endothelial dysfunction via a mechanism involving upregulation of arginase-1 (Arg1), ROS formation, and subsequent impairment of nitric oxide bioavailability (5, 6). These results represent a paradigm shift in our understanding of endothelial dysfunction and shed light on the possibility that factors external to the endothelium — rather than the endothelium per se — are critically involved in vascular damage. Although the crosstalk between RBCs and the endothelium is intriguing, the exact mechanisms by which RBCs impair endothelial function remain poorly understood. In this issue of the *JCI*, Collado et al. show that the detrimental, prooxidant action of RBCs on the vascular endothelium is mediated by extracellular vesicles (EVs) (7). In this study, EVs derived from RBCs of patients with type 2 diabetes (T2D RBC-EVs) were taken up by the endothelium and were able to impair endothelium-dependent relaxation as compared with EVs derived from healthy individuals (H RBC-EVs). Indeed, inhibition of EV uptake by heparin prevented endothelial dysfunction, thus showing that EVs are critically involved in this phenomenon. The detrimental effect of EVs in this setting was driven by an EV-mediated transfer of the prooxidant enzyme Arg1 from RBCs to endothelial cells. Pharmacological inhibition of Arg1 by 2(S)-amino-6-boronohexanoic (ABH) prevented the increase in ROS and the impairment of endothelial vasorelaxation in mouse aortic rings exposed to RBCs from patients with T2D. To further demonstrate the causal role of EV-derived Arg1, the authors used approaches to deplete Arg1 in the endothelium of mice, including siRNA-mediated knockdown and cell-specific deletion from mice with endothelium-specific deletion of Arg1. Notably, treatment with T2D RBC-EVs increased Arg1 protein levels in endothelial cells, thus suggesting an active transfer of exogenous Arg1 from RBCs to the endothelium. Taken together, these results uncover a mechanism underlying diabetic endothelial dysfunction and suggest that biological signals external to the endothelium play an important role in endothelial ROS generation and dysfunction (Figure 1) (7).

## EVs as carriers of prooxidant signals

An interesting observation from this study was that the amount of EVs released from T2D RBCs was lower than in H RBC-EVs, whereas only diabetic RBCs showed an uptake and were able to transfer Arg1 to endothelial cells. A possible explanation for this phenomenon could be the remodeling of EV membrane proteoglycans in diabetic versus control individuals. Indeed, heparin — a naturally occurring glycosaminoglycan — prevented the uptake of T2D RBC-EVs. Consistent with this observation, proteomic analysis of the EV cargo showed the involvement of heparan sulphate proteoglycans, namely syndecan-4 and CD44. A reduced amount of EVs from diabetic RBCs is somewhat surprising, given previous observations linking systemic oxidative stress and hyperglycemia with enhanced EV release from RBCs (8). The study by Collado et al. shows that is not the quantity of the EVs released but rather their quality that determines the rate of uptake by the endothelium (7). Indeed, the release of EVs per se should not be interpreted as a pathological event in RBCs. Human RBCs shed approximately 20% of their membrane area throughout their lifespan, moving from a surface area of 135 μm^2^ into an area of 112 μm^2^ during their 120-day lifespan (9). What should be considered pathological is the rewiring of EVs’ cargo in diabetes, leading to changes in heparan sulphate proteoglycans. A better understanding of proteoglycan-related changes and their role in EV uptake could set the stage for new therapies blocking the transfer of biological information from RBCs to the vascular endothelium (10). Another interesting finding of this study was the relative similarity in Arg1 protein levels between T2D RBC-EVs and those from healthy individuals, suggesting that EV uptake, as opposed to upregulation, drives endothelial damage in this setting.

## Considerations and clinical perspectives

The notion that an external source of Arg1 precipitates endothelial dysfunction is supported by previous work showing that endothelial ablation of Arg1 in mice does not rescue diabetes-induced endothelial dysfunction (11). A possible caveat of the study by Collado et al. (7) relates to the ex vivo experimental setup testing human T2D RBC-EVs on mouse aortic rings. Although this work provides proof-of-concept evidence on the role of RBC-EVs from patients with diabetes, there is a lost opportunity to study the effects of RBC-derived Arg1 in vivo, a condition which better reflects features of the diabetic microenvironment. Further experiments could have included the study of EV uptake by the diabetic endothelium as well as the contribution of hyperglycemia on EV uptake. Albeit Arg1 has proven to be a very interesting molecular target in this setting, other players of the game potentially involved in ROS generation may have been neglected in this study. Indeed, proteomic analysis of EV cargo showed the dysregulation of several ROS-related proteins in diabetic versus control RBC-EVs.

The clinical relevance and impact of the present findings is outlined by the notion that — besides endothelial dysfunction — RBC-EVs are potentially implicated in a plethora of comorbidities clustering with diabetes. For example, RBC-EVs could contribute to explaining the hypercoagulable state observed in T2D patients given the expression of tissue factor and/or a phosphatidylserine surface, which supports the assembly of enzymatic coagulation complexes (12). Yuan et al. revealed increased levels of RBC-EVs in patients with acute myocardial infarction as compared with subjects without coronary artery disease (13). Along the same line, in patients with ST-elevation myocardial infarction (STEMI) treated with primary percutaneous coronary intervention (PCI), RBC-EVs were an independent predictor of adverse clinical events following coronary revascularization (14). A recent experimental study that employed a transgenic mouse model to study the recipient cells of RBC-EVs in vivo showed uptake in several organs including the heart, kidney, lungs, spleen, and brain (15). Matsumoto et al. found that RBCs produce α-syn–rich EVs (in a concentration of approximately 1,000-fold higher than in the cerebrospinal fluid), which can cross the blood-brain barrier and can be taken up by microglial cells, promoting microglial inflammatory responses (16). Given the strong link between α-syn, neuroinflammation, and dementia (17), RBC-EVs could represent an important mediator of brain microvascular and cerebral damage in diabetes, thus promoting an accelerated cognitive decline. Another important aspect to consider is that RBC-EVs are very stable in the bloodstream, and this property amplifies their noxious action on target organs (8).

The findings by Collado et al. (7) open new avenues for potential biomarkers of endothelial dysfunction in patients with diabetes. Several studies have shown that EVs may function as biomarkers in several conditions including cancer and cardiovascular disease (18). However, in the present study, Arg1 was not increased in T2D RBC-EVs as compared with H RBC-EVs, with the main molecular feature of diabetic RBCs being the remodeling of the proteoglycan landscape. Research in this direction could identify biomarkers that reflect EV-cargo remodeling in RBCs from diabetic patients. Furthermore, this study has implications that go beyond diabetes. Transfusion of RBCs is among the most common medical interventions both in emergency and nonemergency settings. EVs are released both by endogenous circulating RBCs and RBCs during storage (19, 20). In this regard, transfusion of RBCs from individuals with cardiovascular risk factors (i.e., individuals over 65, smokers) could foster RBC-EVs’ release in the host with subsequent transfer of biological material and detrimental effects on target organs, including the endothelium.

## Conclusions

Collado et al. (7) advance the field by providing a mechanism and a putative molecular target to prevent endothelial dysfunction in patients with diabetes. Of clinical relevance, heparin was found to impede RBC-EVs’ uptake by the endothelium. Further work is required to study proteoglycan biology and alterations of the EV cargo regulating their uptake by the endothelium. Such findings could lead to the identification of refined glycosaminoglycans specifically blocking EV-mediated transfer of biological signals. Evidence provided by this important study lets us believe that the solution to the problem could not be in the vascular endothelium, but in the blood.

## Version 1 05/15/2025

Electronic publication

## References

[1] Yang DR (2024). Endothelial dysfunction in vascular complications of diabetes: a comprehensive review of mechanisms and implications. Front Endocrinol (Lausanne).

[2] Paneni F (2013). Diabetes and vascular disease: pathophysiology, clinical consequences, and medical therapy: part I. Eur Heart J.

[3] Byrne NJ (2021). Therapeutic potential of targeting oxidative stress in diabetic cardiomyopathy. Free Radic Biol Med.

[4] Goszcz K (2015). Antioxidants in cardiovascular therapy: panacea or false hope?. Front Cardiovasc Med.

[5] Zhou Z (2018). Erythrocytes from patients with type 2 diabetes induce endothelial dysfunction via arginase I. J Am Coll Cardiol.

[6] Pernow J (2019). Red blood cell dysfunction: a new player in cardiovascular disease. Cardiovasc Res.

[7] Collado A (2025). Erythrocyte-derived extracellular vesicles induce endothelial dysfunction through arginase-1 and oxidative stress in type 2 diabetes. J Clin Invest.

[8] Ma SR (2023). Red blood cell-derived extracellular vesicles: an overview of current research progress, challenges, and opportunities. Biomedicines.

[9] Ciana A (2017). Membrane remodelling and vesicle formation during ageing of human red blood cells. Cell Physiol Biochem.

[10] Cerezo-Magana M (2023). Proteoglycans: a common portal for SARS-CoV-2 and extracellular vesicle uptake. Am J Physiol Cell Physiol.

[11] Chennupati R (2018). Deletion of endothelial arginase 1 does not improve vasomotor function in diabetic mice. Physiol Rep.

[12] Noubouossie DF (2020). Red blood cell microvesicles activate the contact system, leading to factor IX activation via 2 independent pathways. Blood.

[13] Yuan Y (2020). Association of endothelial and red blood cell microparticles with acute myocardial infarction in Chinese: a retrospective study. Ann Palliat Med.

[14] Giannopoulos G (2014). Red blood cell and platelet microparticles in myocardial infarction patients treated with primary angioplasty. Int J Cardiol.

[15] Valkov N (2021). SnRNA sequencing defines signaling by RBC-derived extracellular vesicles in the murine heart. Life Sci Alliance.

[16] Matsumoto J (2017). Transmission of α-synuclein-containing erythrocyte-derived extracellular vesicles across the blood-brain barrier via adsorptive mediated transcytosis: another mechanism for initiation and progression of Parkinson’s disease?. Acta Neuropathol Commun.

[17] Forloni G (2023). Alpha Synuclein: neurodegeneration and inflammation. Int J Mol Sci.

[18] Kumar MA (2024). Extracellular vesicles as tools and targets in therapy for diseases. Signal Transduct Target Ther.

[19] D’Alessandro A (2015). An update on red blood cell storage lesions, as gleaned through biochemistry and omics technologies. Transfusion.

[20] Rubin O (2008). Microparticles in stored red blood cells: an approach using flow cytometry and proteomic tools. Vox Sang.

